# Range of instrumentation and complications in the surgical treatment of spinal deformity in patients with Parkinson's Disease

**DOI:** 10.1186/1748-7161-10-S1-P26

**Published:** 2015-01-19

**Authors:** Yasumasa Ohyama, Takahiro Iida, Junya Katayanagi, Ken Mine, Kazuyuki Matsumoto, Hirokazu Furukawa, Takashi Toumura, Satoru Ozeki

**Affiliations:** 1 Department of Orthopaedic Surgery Dokkyo Medical University Koshigaya Hospital, Japan

## Purpose

Parkinson's Disease (PD) patients are a high-risk population in adult spinal surgery and there is no general consensus regarding the surgical correction of spinal deformity in these patients. We perform long fusion for PD spinal deformity in our institution and this study is a retrospective assessment of the radiological and clinical outcomes of posterior spinal fusion with instrumentation for spinal deformity in Parkinson's disease (PD).

## Methods

Between 2007 and 2012 eleven PD patients (1 female and 10 males) underwent corrective spinal surgery at our institution. The mean age at surgery was 67. Spinal scoliosis and kyphosis were treated with posterior correction. 8 patients were treated with instrumentation from the superior thoracic vertebra to the sacrum (superior thoracic group hereafter). 3 patients were instrumented from the inferior thoracic vertebra to the sacrum (inferior thoracic group hereafter). Data examined in this study is from follow-up at a minimum of 1 year (mean=27 months) postoperatively.

## Results

Operative time was an average of seven hours 35 minutes. Perioperative bleeding was an average of 3,900 ml. The length of stay was an average of 65 days. Correction of Sagittal Vertical Axis (SVA), lumbar lordosis (LL), Pelvic Tilt (PT) and C7-coronal center vertical line (C7-CVL) was achieved in all patients. However, in the inferior thoracic group, sagittal radiographs demonstrated progression of PJK, increased SVA, and pelvic tilt between the measurements taken immediately after surgery and those taken at final follow-up. In this group complications such as pseudarthrosis, PJK, cage dislodgement, rod breakage and infection were also noted. Postoperative complications requiring multiple surgeries were encountered in all cases of the inferior thoracic group. By contrast, in the superior thoracic group, although minor complications such as skin ulcers were encountered, posture correction was maintained, postoperative SRS scores remained good, and a high satisfaction rate was obtained without multiple operations.

## Conclusion

Instrumented correction of spinal deformity in PD patients is highly invasive and fraught with complications. However, the contrast observed in results between the two groups in our study seems to support the importance of achieving global sagittal alignment from the pelvis all the way to the upper thoracic vertebra.

